# Influence of footwear fitting on feet morphology in 9 year old girls

**DOI:** 10.1186/s12887-020-02245-z

**Published:** 2020-07-20

**Authors:** Ewa Puszczalowska-Lizis, Paulina Zarzyczna, Wioletta Mikulakova, Mariusz Migala, Slawomir Jandzis

**Affiliations:** 1 Medical College, Institute of Health Sciences, University of Rzeszow, Warzywna 1A Street, 35-959 Rzeszow, Poland; 2 Center of Physical Rehabilitation “Tutmed”, Non Public Health Care Institution, Przemyska 24 Street, Sanok, Poland; 3 Faculty of Health Care, Department of Physiotherapy, University of Presov, Partizanska 1 Street, 080 01 Presov, Slovakia; 4 Faculty of Physical Education and Physiotherapy, Institute of Physiotherapy, Opole University of Technology, Proszkowska 76 Street, 45-758 Opole, Poland

**Keywords:** Children’s feet, Indoor shoes, Footwear fitting

## Abstract

**Background:**

The human foot is shaped throughout all life in a way that is individual for every human being. Footwear fitting in the process of foot development is the issue covered by a limited range of empirical studies. This prompted the authors to undertake this subject of the study aimed at the influence of fitting of regularly worn inside the school footwear on feet morphology in primary schoolgirls.

**Methods:**

The study group comprised 100 girls aged 9. Feet characteristics were recorded by CQ-ST podoscope. The footwear fitting to the feet of the examined girls was tested using the Clevermess device. The data were analyzed based on the Student’s t test, Wilcoxon test and regression analysis.

**Results:**

Appropriately fitted right indoor footwear was worn by 48% of the subjects while the left one by 43% of the group. Appropriate fitting in relation to the left and right foot width was noted in 23% of the group. The statistically significant combined effect of predictors characterizing footwear on the value of Wejsflog index of the right (*p* < 0.001) and left (*p* < 0.001) foot and influence of the length excess on the heel angle of the left foot (*p* = 0.006) were found.

**Conclusions:**

Most examined girls wear poorly fitted indoor footwear. The length excess of the indoor footwear has connections with the Wejsflog index of the right and left foot and the heel angle of the left foot. The larger the length excess, the lower the transverse arch. In the production of indoor footwear the differences in the feet width should be taken into account.

## Background

The human foot is shaped throughout all life in a way that is individual for every human being. The variability of its shape is a result of various factors, including genetic, environmental, socio-economic, lifestyle and type of footwear worn [1–8]. According to Kinz et al. [9] 96–99% of children are born with healthy feet, however, 40% suffer from health problems related to feet in their adult life. The literature in the subject indicates that the reason for this is wearing inadequately fitted shoes during childhood [10–12]. Delgado-Abellán et al. [13] on the basis of the analysis of the foot morphology in Spanish school-aged children emphasized that biological factors such as age and sex, as well as extra-biological factors including the type of footwear worn, influence the foot structure. Mauch et al. [2] found that due to the intensive pace of developmental processes, the selection of appropriate footwear for children is more important than in adults. According to Zhang at al [14]., children’s feet are in the period of skeletal calcification, along with the development of joints, muscles and ligaments. They are an important stage to form stable joints and powerful arches, and also a high incidence of foot deformities. Pre-school and early-school age is of great importance for later foot health and capacity due to the dynamics of developmental changes. Therefore, the child’s foot during this period requires special attention [13–17]. The early school period is associated with a change in lifestyle, the essence of which lies in the transition from free, largely individually regulated child’s effort and rest into being in a sitting position for many hours a day. The type of footwear worn in the school building is also changed. Soft slippers that children used to wear in the kindergarten are replaced by more or less comfortable indoor footwear. Most parents choose rubber-soled shoes that do not slip on the smooth surface of the school corridors and thus minimize the risk of potentially dangerous falls, and additionally have a fashionable and attractive look [18, 19].

Dinato et al. [20] stressed the need to consider differences in the foot structure in primary school children when choosing footwear. Xu et al. [17] observed sex-associated differences in the foot morphology of the school-aged children representing 7 regions in China: North China, Southern China, East China, Central China, Southwest China, Northwest China, and Northeast China. The authors performed measurements of length variables (foot length, medial ball length, lateral ball length, instep length), width variables (ball width and heel width) and height variables (first metatarsal head height, fifth metatarsal head height, arch height, instep height). Girls showed significantly smaller values across all absolute foot measurements compared to boys of the same age, except for the height of the fifth metatarsal head at ages 10 and 12, and the arch height in ages 7–12. Walther et al. [21] emphasized that girls’ feet are more slender, while boys are characterized by more massive metatarsals. According to Yurt et al. [22] the girls’ feet are more often deformed due to more delicate structure. Zhang and Wang [23] stressed that wearing certain styles of shoes may cause discomfort and disturb the normal gait pattern. On this basis, it can be concluded that fashionable footwear does not always meet basic health standards and therefore may cause foot deformation. The authors pointed out that although children’s shoes are intended for children, it is the parents who usually buy them. Therefore, they should demonstrate appropriate knowledge about the rules of choosing the right footwear. It is also worth emphasizing the inter-gender differences of the foot growth rate. In the studies by Xu et al. [17], based on the Asian population, mean growth rates in foot length were 4.3% for boys and 3.9% for girls per year. The largest growth rate occurred at 7–8 and 8–9 years for girls and 8–9 and 10–11 years for boys, and the smallest growth rate occurred at 9–10 and 11–12 years in both girls and boys. Measurements indicating the largest mean increases were arch height, instep length, and heel width for both boys and girls. In turn, results obtained by Delgado-Abellán et al. [13] in school-age Spanish children showed that foot dimensions increase at an average of 3–5% per year and foot length begin to differ between boys and girls at the ages of 8 and 10 years.

This knowledge is of great diagnostic importance and indicates the need for careful monitoring of the condition of the feet, especially in the aspect of footwear fitting. Incorrectly fitted shoes may hinder the development of the feet and cause their deformation. In turn, disturbances in the construction of the feet may have a negative impact on the structure and function of the higher parts of the kinematic chain [12, 24, 25].

The analysis of extensive literature indicates that footwear fitting in relation to the developing foot is the issue with a limited range of empirical studies. Delgado-Abellán et al. [13] show that the variations in foot measurements underwent a gradual increase with age in both boys and girls. Gender differences appeared at the age of 8–9 years, when the girls in the sample group were found to wear smaller shoes than those they should have worn. Most foot dimensions begin to differ between boys and girls at the age of 8 years. According to Szilagyi-Pagowska [26] the age of 9 is the beginning of the prepubescence phase characterized by the acceleration of growth and the dynamic development of self-awareness (gender identity). Girls are beginning to indicate the longing for emphasizing gender in clothing. That is why, compared to boys, they are more likely to follow the fashion, which in many cases means that when choosing shoes, their primary purpose, such as protection of feet against injuries and adverse weather conditions, is no longer important. This was the reason for undertaking the subject of the paper, the aim of which is to analyze indoor footwear fitting and its impact on the feet construction features in 9-year-old girls.

## Methods

### Participants

The study performed in April 2018 comprised 9-year-old girls attending the randomly selected schools in the Sanok administrative district. The sample size representative for the site was estimated in due consideration of 95% confidence level, and a 5% level of admissible error of fraction estimation. This number totalled 134 girls. Preliminary allocation of girls to the so estimated study group was made with the aid of simple dependent drawing method. The selected group was subsequently verified in terms of its compliance with pertinent inclusion and exclusion criteria. The inclusion criteria were: age 9 years; lack of any diseases and/or injuries of the musculoskeletal system; lower limbs with no previous surgical interventions, including feet; no genetically-dependent hallux valgus, as confirmed through an interview. The exclusion criteria: underweight, overweight, or obesity; not understanding the instructions that were necessary for the measurement procedures; refusal to participate in the trial.

Following completion of the allocation procedure, it was established that 34 girls could not take part in the study protocol owing to their non-compliance with the attendant criteria. There were 100 girls enrolled into the study.

The mean body mass of the examined girls was 30.42 ± 6.80 kg, the mean body height was 132.88 ± 6.32 cm, while the average BMI amounted to 17.09 ± 2.82 kg/m^2^.

### Design

The CQ-ST podoscope (manufactured by *Electronic System*) was applied as the research tool of choice. The study protocol entailed the measuring of the plantar feet surfaces in a relaxed stance, with the upper limbs hanging down freely along the body. Body weight was evenly distributed on both feet. Both feet were subjected to an assessment simultaneously. The calculations comprised the following indices:

- foot length – the line D, connecting the most distal point of the forefoot (on the pad of the longest toe) with the farthest point within the hindfoot [cm];
- foot width – the line S, connecting the most medially located point on the head of the first metatarsal bone (metatarsale tibiale, mtt) with the point lying most laterally on the head of the Vth metatarsal bone (metatarsale fibulare, mtf) [cm];
- Clarke’s angle (longitudinal foot arch) – is constructed by drawing a tangent to the medial edge of the foot and the line joining the point of the largest recess of the footprint with the mtt point [°];
- the Wejsflog index (transverse foot arch) – is the ratio of the length to the width of the foot;
- heel angle γ (transverse foot arch) – is comprised between the tangents to the medial and lateral edge of the foot, which cross over the heel [°];
- hallux valgus angle (α) – the angle between the tangent line to the medial edge of the foot and the tangent to the pad of the big toe, derived from the mtt point [°];
- the angle of the varus deformity of the fifth toe (β) – the angle between the tangent line to the lateral edge of the foot and the tangent to the pad of the fifth toe, derived from the mtf point [°].

**Fig. 1:**
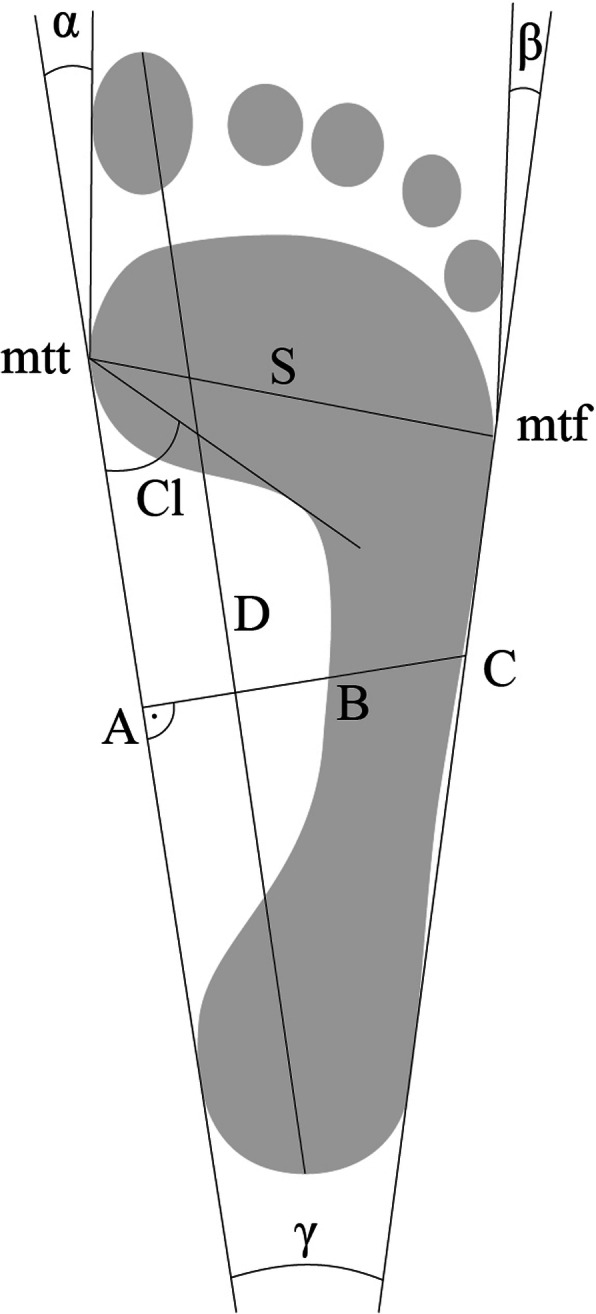
The manner of marking the feet structure indices

The procedures for calculating the feet structure indices are shown in Fig. 1.

The footwear that the children worn inside the school (indoor footwear) were examined. The typical indoor shoes for schoolgirls in Poland are canvas, lace-up sneakers, with a rounded, stiffened, rubber toe and stiffened heel, on a flat rubber sole. The most commonly used method of connecting the shoe sole with the upper part is sticking, thus it can be concluded that this type of footwear is not very durable, and the materials from which it is made may be damaged during their use. Therefore, it should be recognized that this type of footwear requires frequent replacement. The advantage of sneakers is light weight and non-slip sole, ensuring stability.

The weight of the tested footwear was on average 0.22 ± 5.00 dag, and duration of their use ranged from 0.50 to 3.00 months (mean 1.59 ± 0.72 months).

Footwear fitting to the feet of the examined children was tested using the Clevermess device (manufactured by *Clevermess GmbH*), which consisted of a toe limiter, heel stop, buckles to measure foot width, extension mounted on the back of the device, allowing measurements of shoes larger than size 31. The measurement sample covering 100 feet, made by two independent testers confirmed that the reliability of this measuring tool is excellent. The average measurement of the length excess of footwear made by the first tester was 8.99 ± 3.51 (95% confidence interval of the mean, 8.29 to 9.69 mm), mean measurements made by the second tester 8.90 ± 3.39 (8.23 to 9.57 mm), and intraclass correlation coefficient ICC = 0.993. The average measurement of width excess made by the first tester was 3.55 ± 2.61 (4.49 to 4.71 mm), the average of measurements by the second tester 3.53 ± 2.64 (4.42 to 4.47 mm), ICC = 0.992.

First, the length and width of the foot were measured. To measure the length, the child’s foot was placed on the device so that the stops adhered closely to the heel and toes. During the measurement of the foot width, attention was paid that the clamps should be pressed against the width of the foot and were connected over its dorsal surface. The measurement results were automatically saved in the device’s memory. Subsequently, the device was put into the shoe in order to measure the length and width of its interior. After 3 s from placing in the shoe, the device signaled the end of the measurement. After removing the scoop from the shoe, the result was on the screen, which contained information about the free space in the shoe (the length excess and the width excess).

In children during the early school period, the rate of growth is on average 1 cm per year [27], which is why it is assumed that the functional excess, which is the difference between the foot length and the inner length of footwear for children under 15 should be 10 mm [19]. Considering that the acceptable measurement error in the interpretation of our test results is within ±2 mm, correct values of the functional length excess within 8 to 12 mm were assumed. When assessing footwear fitting in terms of length, the following criteria were adopted:

- too short footwear – when the length excess was less than 8 mm,
- appropriate footwear – when the length excess was 8–12 mm,
- too long footwear – when the length excess was over 12 mm [19, 28].

The following criteria were taken into account when assessing footwear fitting in terms of width:

- too narrow footwear – when the width excess was less than 1 mm,
- appropriate footwear – when the width excess was 1–3 mm,
- too wide footwear – when the width excess was over 3 mm [28].

Aditionally, a subjective evaluation of the perceptions of footwear fit was conducted. Participants were asked to report, on 100 mm visual analogue scales (VAS), their perceptions of the shoe fit (using the anchors “poorest fit possible” and “best fit possible”).

In order to preserve the integrity of the research process, all the measurements were taken in the gym, in the morning, using the same measuring instruments operated by the authors. The subjects did not undertake intense physical activity during the 12 h preceding the measurements. Girls wore their gym uniforms. For the podoscopic examination, the girls were without shoes and socks, while for measuring the footwear fitting with Clevermess device, they put on their socks. All study protocol procedures were pursued in full compliance with the Helsinki Declaration. All participants, their parents or legal guardians were furnished with detailed information on the aims and methods to be used throughout the study, and gave their written informed consent to participate in the study.

### Analysis

Based on the data collected, the descriptive statistical calculations were made. Consistency of pertinent variables with reference values in normal distribution was verified by means of the Shapiro-Wilk test. The results for the right and the left foot were compared with Student’s t test or Wilcoxon test. Parametric Student’s t test for dependent samples was applied to analyze the data with normal distribution, while the non-parametric Wilcoxon test was used due to the non-compliance with the normal distribution. The influence of independent variables (predictive, explained as length and width excess of footwear) on dependent variables (featured, such as foot construction) was estimated on the basis of multiple regression analysis and simple regression. The statistical significance was set at *p* < 0.05. The Stat Soft STATISTICA application (version 13.1) was used to process the test results.

## Results

The comparison of the values of podometric indicators obtained for the right and left foot allowed us to recognize that the feet of the studied girls are characterized by symmetry (Table 1).

**Table 1:** Comparison of the values of podometric indicators obtained for the right and left foot in the study subjects

Feature		$\overline{x}$±SD	max–min	Q_25_	Me	Q_75_	t/Z	p
Foot length [cm]	rf	19.54 ± 1.16	22.10–17.20	18.70	19.45	20.40	t = −0.03	0.976
	lf	19.54 ± 1.17	22.10–17.20	18.70	19.50	20.40		
Foot width [cm]	rf	7.43 ± 0.47	8.90–6.50	7.05	7.40	7.70	t = −0,70	0.482
	lf	7.48 ± 0.45	8.50–6.50	7.15	7.50	7.70		
Clarke’s angle [°]	rf	33.38 ± 9.49	50.00–11.00	29.50	35.00	40.00	Z = -0.10	0.920
	lf	32.02 ± 10.21	49.00–8.00	24.00	34.00	40.00		
Wejsflog index	rf	2.63 ± 0.14	3.00–2.33	2.54	2.63	2.72	t = 0.83	0.409
	lf	2.62 ± 0.14	2.89–2.24	2.52	2.61	2.72		
Heel angle γ [°]	rf	17.30 ± 2.00	23.00–13.00	16.00	17.00	18.00	Z = -0.10	0.920
	lf	17.26 ± 1.74	21.00–13.00	16.00	17.00	18.00		
Hallux valgus angle α [°]	rf	5.42 ± 4.47	16.00–0.00	0.50	5.00	8.00	Z = -0.25	0.803
	lf	5.57 ± 4.09	17.00–0.00	3.00	5.00	8.00		
The angle of the varus deformity of the fifth toe β [°]	rf	13.69 ± 5.20	26.00–0.00	10.00	14.00	17.00	Z = 0.04	0.966
	lf	13.31 ± 5.77	27.00–0.00	10.00	14.00	17.00		

*rf* Right foot, *lf* Left foot, $\overline{x}$ Arithmetical mean value, *SD* Standard deviation, *max* Maximum value, *min* Minimum value, *Q*_*25*_ Lower quartile, *Me* Median, *Q*_*75*_ Upper quartile, t value of the Student’s t test statistic, Z value of the Wilcoxon test statistic, *p* Probability value

Data in Table 2 indicate that the longitudinal flatfoot involved 28% of the right and 30% of the left feet, while the right longitudinal hollow feet were found in 2% of the subjects and the left in 1% of the group. The transverse flatfoot was recorded in 24% of the right and 23% of the left feet. The right lateral hollow feet were found in 6% of the girls and the left in 4% of the group. Hallux valgus of the right foot was noted in 18% of the girls and the left in 16% of the subjects. The most frequent deformity was the varus of the fifth toe, which was found in 76% of the right and 82% of the left feet.

**Table 2:** Number of subjects with various types of longitudinal and transverse arch and setting of the hallux and the V toe in the study subjects

	Right foot	Left foot
	%	
The medial longitudinal arch based on the Clarke’s angle - reference values: 34–48° [29]		
Flat foot	28.0	30.0
Normal foot	70.0	69.0
High arched foot	2.0	1.0
Transverse arch based on the heel angle (γ) - reference values: 15–18° [29]		
Flat foot	24.0	23.0
Normal foot	70.0	73.0
High arched foot	6.0	4.0
Setting of the hallux based on the hallux valgus angle (α) - reference values: 0–9° [29]		
Normal setting	82.0	84.0
Hallux valgus	18.0	16.0
Setting of the V toe based on the V toe varus deformity angle (β) - reference values: 0–9° [29]		
Normal setting	24.0	18.0
The V toe varus deformity	76.0	82.0

The mean length functional excess of the indoor footwear was 8.43 ± 3.16 mm (right foot) and 8.35 ± 3.36 mm (left foot). The mean width functional excess of the tested footwear was 3.77 ± 4.22 mm (right foot) and 3.11 ± 5.46 mm (left foot). Values of standard deviations indicate a large variation in the value of the analyzed features. There were no statistically significant differences in terms of the functional excess value of length and width recorded for the right and left feet (Table 3).

**Table 3:** Comparison of the functional excess of indoor footwear in the examined girls

Excess of footwear [mm]		$\overline{x}$±SD	max–min	Q_25_	Me	Q_75_	Z	p
Length	rs	8.43 ± 3.16	17.00–4.00	6.00	8.00	10.00	0.31	0.755
	ls	8.35 ± 3.36	17.00–3.00	6.00	8.00	10.00		
Width	rs	3.77 ± 4.22	14.00–(−6.00)	1.00	4.00	6.50	0.96	0.340
	ls	3.11 ± 5.46	17.00–(−8.00)	−1.00	3.00	7.00		

*rs* Right shoe, *ls* Left shoe, $\overline{x}$ Arithmetical mean value, *SD* Standard deviation, *max* Maximum value, *min* Minimum value, *Q*_*25*_ Lower quartile, Me Median, *Q*_*75*_ Upper quartile, *Z* Value of the Wilcoxon test statistic, *p* Probability value

On the basis of the measurement of the length excess of the footwear, it was found that appropriately fitted right indoor footwear was worn by 48% of the subjects while the left one by 43% of the group. Too short right footwear was found in 40% of the group and the left one – in 43% of the group. Too long right and left footwear had respectively: 12% of the group and 14% of the group.

On the basis of the measurements of the width excess of the footwear, we found that an appropriate fitting in relation to the left and right foot width was noted in 23% of the group. Too wide right shoe had 55% of the group and the left one – 47% of the group, while too narrow right shoes wore 22% of the group and the left one - 30% of the group (Table 4).

**Table 4:** Passivity of indoor footwear in the study subjects

Footwear fitting		Right shoe (*n* = 100)	Left shoe (*n* = 100)
		%	
Length	Too short	40.0	43.0
	Appropriate	48.0	43.0
	Too long	12.0	14.0
Width	Too narrow	22.0	30.0
	Appropriate	23.0	23.0
	Too wide	55.0	47.0

Participants reported very favourable perceptions of indoor footwear fit, with scores ranging from 84 to 100 mm (mean VAS = 94.50 ± 0.55 mm).

Table 5 presents structural parameters of multiple regression models and simple regression. Multiple regression models with two explanatory variables (length and width excess of footwear) of Clarke’s angle variance were statistically insignificant. Also, the analysis of simple regression results did not show statistically significant influence of these predictors on the dependent variable.

**Table 5:** Multiple regression models and simple regression, in which independent variables are excess of indoor footwear, while dependent variables are features of the foot structure

Excess of footwear	Multiple regression				Simple regression		
	R	R^2^	F	p	b	r_p_	p
Summary of dependent variable regression: Clarke’s angle of the right foot [°]							
Length	0.05	0.00	0.15	0.864	0.03	0.01	0.925
Width					0.11	0.04	0.689
Summary of dependent variable regression: Clarke’s angle of the left foot [°]							
Length	0.10	0.01	0.52	0.591	0.05	0.01	0.878
Width					0.17	0.08	0.419
Summary of dependent variable regression: Wejsflog index of the right foot							
Length	0.41	0.17	10.37	< 0.001*	−0.02	−0.39	< 0.001*
Width					0.00	0.07	0.468
Summary of dependent variable regression: Wejsflog index of the left foot							
Length	0.46	0.21	13.02	< 0.001*	− 0.01	− 0.43	< 0.001*
Width					0.01	0.37	< 0.001*
Summary of dependent variable regression: heel angle (γ) of the right foot [°]							
Length	0.16	0.03	1.26	0.285	0.07	0.09	0.326
Width					0.03	0.05	0.591
Summary of dependent variable regression: heel angle (γ) of the left foot [°]							
Length	0.32	0.10	5.38	0.006*	0.19	0.31*	0.001*
Width					−0.04	−0.13	0.194
Summary of dependent variable regression: hallux valgus angle (α) of the right foot [°]							
Length	0.13	0.02	0.88	0.414	0.17	−0.12	0.204
Width					0.13	0.10	0.320
Summary of dependent variable regression: hallux valgus angle (α) of the left foot [°]							
Length	0.12	0.02	0.73	0.480	0.16	0.12	0.254
Width					−0.07	−0.09	0.363
Summary of dependent variable regression: the angle of the varus deformity of the fifth toe (β) of the right foot [°]							
Length	0.21	0.05	2.36	0.098	−0.00	− 0.00	0.986
Width					0.27	0.19	0.066
Summary of dependent variable regression: the angle of the varus deformity of the fifth toe (β) of the left foot [°]							
Length	0.16	0.03	1.35	0.262	0.31	0.16	0.116
Width					−0.04	−0.04	0.698

*R* – coefficient of multiple correlation; R^2^ – coefficient of determination; F – the Fisher-Snedecor test; b – the slope of the regression line; r_p_ – partial correlation; p – probability value

* *p* < 0.05

The total impact of predictors characterizing indoor footwear on the value of the Wejsflog index was statistically significant for the right (F = 10.37; *p* < 0.001) and left foot (F = 13.02; *p* < 0.001). Taking into account the right foot, the predictors explained a total of 17% of the variance of the dependent variable (R^2^ = 0.17), and in the case of the left one 21% of the variance of the dependent variable (R^2^ = 0.21). The analysis of simple regression results, taking into account the exclusive influence of each of the explanatory variables on the dependent variable, showed that in the case of the right and left shoes, the size of the length excess has a statistically significant impact on the Wejsflog index. The slope coefficient of the regression line for these variables was b = − 0.02 and b = − 0.01, respectively. This means that the increase in the length excess by a unit will affect the reduction of the Wejsflog index by 0.02 (right foot) and 0.01 (left foot). The size of the width excess of the left indoor shoe had a statistically significant impact on Wejsflog index. The slope coefficient of the regression line for these variables has reached the value of 0.01. This means that the increase in the width excess by a unit affects the increase in Wejsflog index by the value equal to 0.01.

The influence of prediction variables on the heel angle (γ) of the left foot was statistically significant (F = 5.38; *p* = 0.006). These variables jointly explained 10% of the variation of the heel angle (R^2^ = 0.10). Simple regression showed that only the influence of the length excess of the indoor footwear on the heel angle of the left foot was statistically significant (r_p_ = 0.31; *p* = 0.001). The slope of the regression line for variables: the length excess of the indoor footwear and the heel angle of the right foot was b = 0.19, which means that if the shoe length increases by a unit, then the heel angle will increase on average by 0.19°.

Models of multiple regression with two explanatory variables of hallux valgus angle (α) variation were statistically insignificant. Also, the multiple regression model with two variables explaining the angle of the varus deformity of the fifth toe (β) was statistically insignificant.

## Discussion

The issue of the incidence of deformity in children’s feet has been repeatedly undertaken. The authors mostly focused on assessing the longitudinal arch and analyzing potential factors that could lead to its disturbances. In the literature, divergent results regarding the frequency of deformation and the type of factors that may disturb delicate foot structure can be found [3, 16, 24] . Analysis of the available literature also indicates a small number of studies on the impact of footwear on the formation of children’s feet in the early school period. Hettigama et al. [12], on the basis of surveys of school students in Sri Lanka aged 8 and 12, stated that the dynamics of the growth of longitudinal features, including the foot length indicates the necessity of frequent change of shoes, which primarily allows correct fitting to the current foot construction. The authors found that the awareness of the need to wear the correct footwear would counteract diseases and deformities of the feet. They emphasized the urgent need to design and manufacture ergonomic footwear and pointed to the necessity of adaptation of the range of footwear produced by companies to the individual needs of customers, including children in Sri Lanka. The precise selection of footwear should be based on accurate tests, taking into account both foot anatomy as well as the specificity of the gait.

A particularly important issue is fitting a shoe to the individual characteristics of the feet. According to Branthwaite et al. [11], even slight errors in foot circumference and length measurement affect the incorrect shoe fitting. Mauch et al. [2] emphasized that the wrong size of the shoe causes foot injuries and deformities as well as pain. Wolf et al. [1] showed that most of the deformities arise as a result of wearing improperly fitted boots, less often due to the use of wrongly fitted sandals or slippers.

The measure of the degree of footwear fitting to the foot is a functional excess, which on the one hand is a reserve for growing feet, on the other it secures the free space in the footwear needed to lengthen the longitudinal arch (the so-called apparent increase in the length of the foot) during movement or load. The functional excess should take into account the size of the mean foot growth in the period from 6 to 12 months. The results yielded by the present study reveal that adequately fitted indoor footwear had less than half of the girls, while too short shoes had 40% of the subjects. The rest of the girls wore too long shoes. In turn, Klein et al. [18] assessed the quality of footwear worn by Austrian pre-school children aged 3 to 6.5. To measure the length of the inside of the shoe, the authors used a sliding measure. In the classification of the results, they included a travel space of at least 10 mm (optimally 12 mm) more than the length of the foot. Most of the children did not have indoor shoes, or they wore indoor shoes that were not adequately fitted and of worse quality than outdoor footwear. Only 23% of 858 studied children wore well-fitted outdoor footwear, and 9% had well-fitted indoor footwear. Teachers reported that in many cases these shoes were not exchanged throughout the school year. According to the authors, this may be partly due to the fact that parents pay less attention to the footwear that children wear at school, than to the outdoor footwear.

It is equally important to adjust the footwear width, mainly due to the comfort of its use. Chaiwanichsiri et al. [8] pointed out that wearing too narrow shoes, especially in childhood, causes abnormal tension in the muscles and tendons of the feet, the formation of bunions and unsightly thickenings. Too narrow shoes compress the feet, hindering their natural development, as well as impede the work of the muscles. They cause distortion of the toes and stretching of the ligaments maintaining the transverse arch. In turn, wearing too wide shoes is the cause of instability during locomotion and resulting injuries. In our study appropriate fitting in terms of width was found only in 23% of the girls. More than half of the girls wore too wide shoes, and too narrow shoes were worn by 22–30% of the group. Obtained data indicate that in terms of width, indoor footwear was poorly adjusted to the feet of the surveyed girls, despite the fact that the participants reported very favourable perceptions of footwear fit. Such a discrepancy in the objective and subjective assessment of footwear fitting may result from the fact that the foot adjusts to the wrongly selected footwear. The wrong positioning of the foot in the shoe initially does not cause discomfort, but over time affects the formation or deepening of deformation and leads to microdamage of its delicate structures. It is noteworthy that 40–43% of girls wore too short shoes, while 55–47% of them wore too wide shoes. This suggests the need to raise children and, above all, their parents’ awareness of the rules for the selection of footwear, as well as the need to constantly monitor its condition so that in the event of disorders, immediate replacement is made. Since this issue is occasionally undertaken in the literature of the subject, these results are difficult to refer to other reports.

An interesting issue is the impact of the size of the functional excess of footwear on the characteristics of the feet. It is worth paying attention to the statistically significant influence of the length excess of the indoor footwear on the Wejsflog index of the right and left foot and on the heel angle of the left foot. In our material it was found that the larger the length excess, the lower the transverse arch. In addition, the results obtained confirm the previously observed poor adjustment of the indoor footwear to the feet of the surveyed girls. They suggest that, in order to avoid discomfort, children with a wider forefoot wear longer shoes. Obtained results bring concerns whether the shoe lasts used for the production of the analyzed footwear have appropriate proportions of width. Also Delgado-Abellán et al. [13] noted that problems with shoe fit are related to width because shoe designs are usually based only on length. The girls studied wore too short shoes, probably as a result of striving to better fit their narrow feet. It also points to the fact that in the production of footwear, which most often serves indoor, the differences in the width of girls’ feet are not taken into account.

According to Chaiwanichsiri et al. [8] wearing too short shoes can be a direct cause of toe deformation. Klein et al. [18] assessed the validity of the claim, which assumes that wearing shoes of insufficient length directly affects the risk of lateral toe dislocation. Out of 1579 respondents, only 24% of children had a correctly set toe, and the remaining feet had hallux valgus. The authors noted a statistically significant relationship between the adequate shoes fitting in terms of length and the hallux valgus angle, which means that the shorter the shoe, the greater the lateral inclination of the first toe. Based on this, it was found that the risk of hallux valgus increases by 17% when wearing shoes too small by 1 size, by 37% if the shoes are 2 sizes too small, and 61% in the case of shoes too small by 3 sizes. In our material, hallux valgus of the right foot was reported in 18% of the girls and the left one in 16% of the subjects. The analysis of multiple regression models allowed us to rule out the influence of predictor variables, such as the length excess and the width excess on the hallux setting.

Much encouraging results, as yielded by the present study, merely make a contribution to further research into this subject, indubitably required with a view to investigating the already established trends even more comprehensively. The present findings would obviously gain even more credence, when confronted with the ones obtained from a much larger population sample, in due consideration of different age groups. Given overall gravity and sheer scale of the issue under study, any subsequent reports would appreciably contribute to its highlighting, while at the same time granting it due prominence in research, and much overdue public exposure.

## Conclusions

Most of 9-year-old girls wear poorly fitted indoor footwear, including approximately 40% of girls wearing too short shoes, while about 50% of them wear too wide shoes. The length excess of the indoor footwear has connections with the Wejsflog index of the right and left foot and the heel angle of the left foot. The larger the length excess, the lower the transverse arch. In the production of indoor footwear the differences in the feet width should be taken into account.

### Limitations of the study

The way the subjects were admitted into the study pursued in line with the adopted inclusion criteria, on the one hand, allowed to ensure homogeneity within a group fully corresponding to pertinent characteristics of the 9 year old population of girls residing in the Sanok administrative district, while on the other affected the restriction of the study range to one area and age group which might well be regarded as a study limitation.

## Data Availability

The datasets generated during and/or analysed during the current study are available from the corresponding author on reasonable request.

## Abbreviations

mtt: Metatarsale tibiale
mtf: Metatarsale fibulare
